# Implementing a Spanish Wikipedia elective for medical students

**DOI:** 10.5195/jmla.2026.2237

**Published:** 2026-04-01

**Authors:** Juli McCarroll

**Affiliations:** 1 juli.mccarroll@wmed.edu, Assistant Professor, Department of Medical Library, Western Michigan University Homer Stryker M.D. School of Medicine, Kalamazoo, MI

**Keywords:** Spanish, medical students, medical education, medical school, curriculum, Wikipedia, consumer health, public health, global health, health information seeking, crowdsourcing, open educational resources

## Abstract

**Background:**

Individuals seeking health information often turn to the Internet for answers. Wikipedia is a dynamic, crowdsourced encyclopedia and one of the most accessed online sources for this content. However, the Spanish Wikipedia is not nearly as in-depth as the English version, creating a large disparity. Medical students with English and Spanish proficiency possess a distinct skill set that positions them to contribute timely, trusted, evidence-based content to the platform and reduce this inequity.

**Case Presentation:**

This case study presents the implementation of a credit-bearing Spanish Wikipedia translation elective by the library for fourth-year medical students at Western Michigan University Homer Stryker M.D. School of Medicine, currently the only Spanish Wikipedia elective in a medical school in the United States. The purpose of the course is to increase the quality and readability of medical articles in the English and Spanish versions of the online encyclopedia using evidence-based medicine (EBM) principles.

**Conclusions:**

The output from this elective demonstrates that medical students can use their medical knowledge and skills to create and improve articles in English and Spanish on Wikipedia and disseminate evidence-based information to millions of consumers worldwide seeking reputable health information. Learners can leverage their specialized training to minimize the gap between these versions and become active participants in global health. By using technology to their advantage, they provide enduring health information that impacts and reaches many more people in a virtual setting than in a traditional one-on-one clinical encounter.

## BACKGROUND

When people need information about health-related topics, they seek answers from healthcare providers, friends and family, advertisements, social media, and search engines [1]. The internet is a fast and convenient way for anyone, including medical students, physicians, and the public, to access health information. This is especially true for individuals that experience barriers to healthcare and have unmet health needs [2,3], including those with limited English proficiency who may experience language discordance with the providers in their area. This linguistic disparity becomes evident when analyzing census data. Although Hispanic people comprise nearly 20% of the population of the United States [4] and an estimated 12% of the US population speaks Spanish at home [5,6], only 6% of physicians identify as Hispanic, and 2% of non-Hispanic providers speak Spanish [7]. Of those providers who identify as Hispanic, data is not available for the number who speak Spanish [7]. There is a large discrepancy between the number of Spanish-speaking patients and providers which creates a need for resources that address and reduce these language barriers. Fortunately, most health information seekers have access to Wi-Fi, if not at home, then at work or in community spaces such as parks, restaurants, libraries, places of worship, and public transportation. Thus, the Internet can play a crucial role in mitigating linguistic divides, especially for Spanish-speakers, who use online medical resources three times more often than English-speaking, adults [8].

When multilingual individuals request information from a health sciences practitioner, it is often taken from two sources that offer patient-facing medical information in languages other than English, including MedlinePlus, which has websites in English and Spanish [9,10], and UpToDate [11]. The first is a free service administered by the National Library of Medicine, which is part of the National Institutes of Health, and provides a medical encyclopedia and information on genetics, health topics, medical tests, medications, and supplements. The second is a paid, subscription-based point-of-care tool, and while the majority of its core clinical topics are in English, some patient education materials are available in Spanish, as are searching and navigation.

On the other hand, when patients look for information online instead of from a provider, they often turn to Wikipedia, which is one of the most highly-accessed resources external to medical databases. It is a free, online, multilingual, openly editable encyclopedia available in nearly 350 languages, with the English version being the largest. It contains nearly seven million articles and received 130-billion-page views in 2024 [12]. A survey of health professionals revealed that 16% had edited a Wikipedia article, while 67% of their patients had consulted the website for health information [13]. This demonstrates the clear need for easily accessible online medical information on Wikipedia. Owing to its size and popularity, it can serve as a bridge between the public and reliable health resources. Although comprehensive, some articles are incomplete, mistranslated, or outdated, contain misinformation or jargon, are written at grade levels that make them difficult to understand, or have references that are behind paywalls; therefore, they could benefit from updating and editing [14–16].

The Spanish edition is not as robust as the English edition, with over two million articles and nine-billion-page views in 2024 [17]. The large difference in the number of articles on the English Wikipedia versus the Spanish Wikipedia indicates that the resource is highly skewed toward articles in English. However, despite its smaller size, the Spanish Wikipedia is likely as important as the English version for consumers looking for answers to their medical inquiries [18].

Medical students have specialized training in the Five As of Evidence-Based Medicine (EBM), which Sackett defined as the “conscientious, explicit, and judicious use of current best evidence in making decisions about the care of individual patients” [19]. This includes asking a clinical question; acquiring evidence from reputable sources and appraising its validity, reliability, and applicability; applying the evidence to make informed decisions; and assessing and adjusting as needed [20]. Therefore, learners are uniquely positioned to contribute up-to-date, evidence-based content to this public health platform and to bridge information gaps. They do this by searching biomedical databases and retrieving relevant citations and critically appraising and applying the literature. As a result, they improve the language, layout, and content of the encyclopedic entries.

When discussing Spanish-speakers in medical education and research, it is important to note that while the term Hispanic commonly refers to people who speak Spanish or are descended from any Spanish-speaking country, Latino describes those with ancestral ties specifically to Latin America. Although distinct, they are often conflated or used interchangeably [21] These diverse origins result in the use of Spanglish and a large number of dialects, accents, and regionalisms and currently there are no recommendations for whether or how these should be incorporated into medical language education [22].

However, students can learn and use a core set of medical terms and phrases that are common and understandable across different regions, providing a useful resource that the majority of Spanish speakers will understand. Although language variations exist, it is not possible to address them all in a single elective, but learners often have multiple Spanish experiences during medical school that will expose them to diverse linguistic and cultural contexts.

## CASE PRESENTATION

### Implementation

In 2018, a leader at Western Michigan University Homer Stryker M.D. School of Medicine approached the library director about the possibility of creating a flexible, credit-bearing elective that could be completed virtually, particularly for students who were off cycle or travelling for residency interviews. The leader and library director were familiar with WikiProject Medicine (WPM), a group formed by health professionals in 2004 to improve medical pages on Wikipedia. It works in conjunction with WikiEdu, a non-profit organization that helps advance the relationship between academia and Wikipedia. The director responded to this request by reaching out to WPM to gather details about running a course at the school and implemented the English version, which ten students completed during the 2018-2019 academic year.

In 2019, after a successful first year, some learners talked to the library director about the need for a similar course in Spanish. They had already taken other Spanish electives at the school and wanted to enroll in more, so the library director reached out to WPM and explored the feasibility of offering the elective in Spanish. With guidance from WPM staff and a bilingual medical student, the WikiProject Medical Translation-Spanish elective was adopted, which is available to all fourth year students at any time during the academic calendar. Although there have been thousands of Wikipedia editing courses in English in the undergraduate setting, there have only been a handful in medical and health professions schools, fewer in Spanish, and currently this is the only Spanish course at a medical school based in the United States. [23–27].

### Objectives

The library director took guidance from the Liaison Committee on Medical Education standards described in the Functions and Structure of a Medical School document to create the course objectives, particularly Standard 6: Competencies, Curricular Objectives, and Curricular Design, and Standard 7: Curricular Content, including Communication Skills and Structural Competence, Cultural Competence, and Health Inequities [28]. Considering this direction, three objectives were developed: improving written communication skills and the ability to translate documents into plain language; identifying the appropriate Spanish vocabulary and grammar for a certain health condition and setting; and evaluating consumer health information for readability and bias.

### Syllabus

The WikiEdu learning management system (LMS) has a built-in weekly timeline for students to follow, listing the tasks that need to be completed each week. The content of the online timeline was converted to a PDF copy to be filed with Educational Affairs and for students who preferred this format over the digital version. It was tailored to include information specific to the school, such as the course director’s contact information, course objectives, and grading criteria.

### Training

A bilingual librarian took over the role of course director for the elective and prepared by completing training provided by the LMS. It included working through the Instructor Orientation Modules and the Student Training Modules; viewing videos about how to create or edit content; reviewing templates for choosing, drafting, and evaluating an article and compiling a bibliography; and viewing guides on editing, evaluating, and illustrating Wikipedia, which required a time commitment of approximately ten hours.

### Selecting a Page to Create or Edit

Approximately 300 active WPM editors continuously work to improve health-related pages and provide lists of articles that need to be updated or expanded [25]. Health-and medical-related articles maintained by WPM contain more content and links, are viewed more often, and are viewed for longer periods than other Wikipedia articles [29]. Consequently, the work that students put into these entries has a relatively high pay-off. During the 4-week course, students chose one of these or selected a topic of interest and located a primary article to create or edit in Spanish or to translate from English to Spanish or the reverse. In addition, they also performed light editing of a secondary article. The course director spent approximately one hour per week supporting and assisting learners.

### Grading

Course directors decide how to grade student work, and WikiEdu provides a sample rubric that was used and can be copied or customized as needed [30]. Assessment was based on improving the content of the primary and secondary articles by including sources of high-level evidence and improving the readability of the articles so that they were aligned with the health literacy level of most consumers, regardless of native language. Since overall health literacy data is generally not broken down by native language, average levels of are used. With respect to readability, since most Americans read at the 6^th^ grade level [31], learners avoided jargon and wrote in plain, lay language whenever possible. Students used an online English or Spanish readability calculator [32,33] or the Flesch-Kincaide Grade Level Formula built into Word to determine readability statistics and the reading level of their article and adjusted it if needed. Grading was based on five components: completing the Wikipedia training modules-15%, drafting the article-20%, evaluating the article-15%, editing the secondary article-20%, and finalizing the article and moving it to the live version of Wikipedia-30%.

## DISCUSSION

### Outcomes

From statements that learners made during weekly check-ins and the work they completed on the dashboard, it was apparent that completing the elective helped improve their written communication skills and their ability to translate health information into plain language for consumers and potential patients. As they progressed through the course, they became better able to identify appropriate Spanish vocabulary and grammar for a given health condition and to identify issues of readability and bias when critically evaluating medical information written for consumers. This helped expand their cultural humility, or self-awareness and a commitment to ongoing learning and growth with respect to other cultures, which should allow them to communicate more effectively and build and strengthen relationships with their future Spanish-speaking patients, families, providers, and staff.

The work that the students put forth and the reliable health information they provided on their pages during the one-month course are long-lasting and have the ability to reach and impact an exponentially larger number of people than they would eventually be able to see in their practices in the future. Table 1 presents a summary of the figures associated with the primary page created or edited by each of the ten students who completed the elective from 2019-2025. Numbers were provided by and pulled from the WikiEdu LMS, which stated that the added words were estimates and may not be exact. In total, learners added 35,359 words and 245 references to their primary articles, and their pages were viewed 17,773 times in the past 30 days as of April 3, 2025. Although pre-data was not collected to measure page improvement comparisons during these academic years, it will be recorded in the future.

**Table 1:** Summary of Wikipedia edits.

Student	Words added to primary article	References added	Page views in the past 30 days
1	4,020	38	5,492
2	2,022	16	2,527
3	13,811	75	369
4	81	14	2,307
5	4,877	19	615
6	2,051	12	292
7	1,720	14	806
8	2,498	19	1,248
9	3,427	30	1,728
10	852	8	2,389
Total	35,359	245	17,773

### Course Director Reflections

In the first check-in via Teams, observations revealed students’ motives for enrolling in the elective which were similar those of other researchers who write about the growing inequality between the number of Spanish-speaking patients and providers that find increased patient satisfaction and outcomes when physicians communicate in Spanish [34]. The course director witnessed a strong social obligation to use their medical training and bilingual language capacity to engage in global health and make health information more available to the public [7,35,36]. She also sensed that due to the Internet's immediate reach and impact, this platform would facilitate the dissemination of information to a wider audience in a significantly shorter time versus waiting until they completed their medical degrees and could communicate with patients in the more traditional in-person or telehealth appointment settings.

During the first check-in, the course director also noted learners’ attitudes and reservations about the course. Most of them had not edited Wikipedia before and seemed to be doubtful that they would be able to effectively use the wikicode markup language to successfully create and improve articles and make them live on the site. It became apparent that they wondered if there was too much to learn in a short amount of time and whether the challenge and difficulty would be worth it. There was also a risk that they might accidentally plagiarize if they did not use the technology correctly to link to the source material and that it would be harder than anticipated to write about medical topics in lay language.

In subsequent check-ins, it became clear that they found it difficult to strike a balance between making articles more comprehensive and keeping them at an appropriate reading level and to find open-access resources. However, by referring back to the tools and training provided by WPM as often as needed and with guidance and assistance from the course director, students were able to successfully create or edit a page on Wikipedia and pass the elective.

Overall, it appeared that learners were able to overcome their initial uncertainties and nearly all of them experienced the same outcomes as others across the country who had taken a Wikipedia course, including increased confidence in locating information gaps, searching biomedical databases, and searching for and selecting trustworthy literature [37]. They seemed to appreciate that the format could be completed virtually, at their own pace, wherever they happened to be, and at a time that was convenient for them. In addition, the work required each week was evenly distributed, so they knew how to budget their time accordingly. Finally, as students in Wikipedia electives at other schools have reported, they demonstrated an appreciation for using their knowledge to contribute to public health on a global scale and gave the impression of satisfaction at being able to see their work available online and track their page views in real time [7,35–38].

The course shell in the LMS and the additional resources that WikiEdu and WPM have created made this elective simple to administer and worth the effort given the students’ large amount of content added, its enduring presence, and the number of page views they will receive. The content remains the same each time the elective is administered, so there is no additional time commitment required during additional terms. The course provided a unique opportunity for bilingual course directors to combine their interests in medical education and Spanish for the benefit of learners and the health information-seeking community. Monolingual instructors can consider implementing the English equivalent of the elective.

Registration has remained fairly low due to several factors. The medical school curriculum is dense and has many required elements, whereas electives are optional, so students may choose to take other electives that are offered, or none at all. In addition, the school has Academic Distinction Programs in specialized areas that students can pursue, which require additional work hours, decreasing the time available for electives. Finally, some students may underestimate their Spanish language ability and do not enroll.

### Future Considerations

WikiProject Medical Translation-Spanish has been well received by students and has resulted in the dissemination of reputable health information worldwide. Going forward, schools might consider offering the course in more of the world’s most-spoken languages to take advantage of learners’ multilingual abilities and their desire to provide trustworthy health information to a broader audience. Although there have been a handful of Wikipedia medical translation courses in languages other than Spanish, they were not in United States-based medical schools and were not recent [39,40].

Some research supports Wikipedia entries and mentions as scholarly activity for faculty, equivalent to peer-reviewed articles and book chapters, with page views being a comparable metric to journal impact factor [41,42]. Schools could accept contributions to this community resource as a publication type for promotion and tenure and include them on their faculty profile pages. Students and faculty can include them in their curriculum vitae and social media pages, such as LinkedIn. Although online encyclopedia entries may be less formal and structured than other traditional publication types, they can impact consumers and patients more directly and thus merit inclusion. In addition to Wikipedia, other social media alternative article-level metrics (alt-metrics), such as mentions and citations that could be counted as academic output, include content on blogs, Facebook, news, and Twitter [16,42,43]. Given the highly online and networked nature of research today, including non-peer-reviewed scholarly content makes sense and would provide a broader and more inclusive representation of publication activity and value compared with the traditional, more restrictive model.

## CONCLUSION

Unlike print encyclopedias of the past, Wikipedia is constantly evolving and relies on editors to create and update its content for consumers worldwide. Editors do not have to be well-known or eminent figures in their fields of study. Rather, they need the requisite subject expertise and desire to advance global health. The impact and reach of this reputable content are crucial for bridging the gap between the public and the health information they seek to access. Implementing this elective and highlighting students’ ability to provide reputable consumer health information has created a symbiotic relationship between learners and consumers, where the former strengthen their EBM skills by increasing the quality and readability of medical articles, and the latter are beneficiaries of high-quality, vetted medical information.

## Data Availability

No data are associated with this project.
